# Comparison of the accuracy and reliability of ChatGPT-4o and Gemini in answering HIV-related questions

**DOI:** 10.1186/s12879-025-12022-x

**Published:** 2025-11-17

**Authors:** Muhammet Salih Tarhan, Meryem Sahin Ozdemir

**Affiliations:** 1 Department of Infectious Diseases and Clinical Microbiology, Mardin Training and Research Hospital, Mardin, 47100 Türkiye; 2 Department of Infectious Diseases and Clinical Microbiology, Basaksehir Cam and Sakura City Hospital, Istanbul, 34480 Türkiye

**Keywords:** AIDS, Artificial intelligence, ChatGPT, Gemini, HIV

## Abstract

**Background:**

Large language models (LLMs) such as ChatGPT and Gemini are increasingly being used to obtain health information, including topics such as HIV. This study aims to comparatively evaluate the accuracy, reliability, and reproducibility of ChatGPT and Gemini in answering HIV-related questions obtained from official public health sources, clinical guidelines, and social media.

**Methods:**

A total of 156 HIV-related questions were asked to ChatGPT-4o and Google Gemini 1.5 Flash across three categories: questions derived from the United States Centers for Disease Control and Prevention (CDC) resources (44.2%, *n* = 69), guidelines (30.8%, *n* = 48), and social media (25.0%, *n* = 39). Responses were rated on a 4-point scale (1 = completely wrong, 4 = completely correct) by two infectious disease specialists. The reproducibility of both LLMs was also evaluated.

**Results:**

The median score (IQR) of the answers generated for all questions was 4.00 (0.00) for ChatGPT and 4.00 (1.00) for Gemini (*p* = 0.051). The rate of completely correct answers was 81.4% for ChatGPT and 71.8% for Gemini (*p* = 0.045). ChatGPT demonstrated significantly lower accuracy in guideline-based questions (47.9%) than in CDC-related (97.1%) and social media-derived (94.9%) questions (*p* < 0.001 for both). Similarly, Gemini demonstrated significantly lower accuracy in guideline-based questions (35.4%) compared to CDC-related (88.4%) and social media-derived (87.2%) questions (*p* < 0.001 for both). Considering the questions according to the topics, the lowest accuracy rate for both LLMs was in the subject of ‘Prevention and Treatment’ (67.2% for ChatGPT, 54.7% for Gemini). The reproducibility of the answers was 94.8% for ChatGPT and 90.3% for Gemini.

**Conclusion:**

ChatGPT and Gemini, answered CDC- and social media–based questions with high accuracy. However, both LLMs showed lower accuracy for guideline-based and “Prevention and Treatment” questions. These findings suggest that while such models may provide useful general information, they are not yet reliable for clinical decision-making, and their outputs should be verified against evidence-based clinical guidelines.

**Supplementary Information:**

The online version contains supplementary material available at 10.1186/s12879-025-12022-x.

## Background

Human Immunodeficiency Virus (HIV) remains a significant global public health challenge. According to the latest UNAIDS estimates for 2024, approximately 40.8 million people were living with HIV globally, and 1.3 million people became newly infected during the year. Approximately 630.000 people died from AIDS-related illnesses, and 31.6 million people were accessing antiretroviral therapy [1]. While life expectancy for people living with HIV has significantly improved due to highly effective antiretroviral therapy, there has been little progress toward eradicating the virus [2]. This suggests that HIV will continue to be a significant public health problem. People living with HIV may have many questions about different topics regarding the disease during this long association. In addition, people seeking to protect themselves against HIV try to get information about many prevention topics, particularly transmission methods and protective measures. Those unable to access healthcare professionals for such questions may instead turn to artificial intelligence (AI).

As AI applications become more widespread, their roles in social life are also expanding. One of these areas is health services. Moreover, AI has the potential to bring about significant developments in the healthcare field [3]. The objective of AI applications is to process extensive datasets through continuously evolving algorithms to provide useful and contextually relevant outputs [4]. Among these applications, large language models (LLMs) enable individuals to obtain information related to diseases and preventive measures quickly. However, such outputs reflect probabilistic language predictions rather than verified medical guidance. Consequently, individuals may pose a variety of health-related queries and interpret the responses without assuming they constitute definitive medical advice. LLMs such as ChatGPT (Chat Generative Pre-trained Transformer; OpenAI, San Francisco, CA, USA) and Gemini (Google, Mountain View, CA, USA) are among the most frequently used AI tools [5, 6]. These LLMs predict the most likely next word based on patterns learned from vast amounts of text—rather than directly retrieving or transferring factual information. Nevertheless, the accuracy and reliability of the health-related information they provide remain topics of ongoing debate. Although several studies have explored the use of large language models (LLMs) in the context of HIV, this remains a relatively limited area of research. Most existing studies have focused on general or educational content, leaving uncertainty about the accuracy of LLM-generated responses to guideline-based questions. Therefore, this study aimed to comparatively assess the accuracy, reliability, and reproducibility of responses generated by two widely used LLMs -ChatGPT and Gemini- on HIV-related questions.

## Methods

The questions were categorized into three main groups. In the first group, the questions were directly obtained from the Centers for Disease Control and Prevention (CDC) “Questions and Answers for the Public” section. In the second group, the questions were derived from the European AIDS Clinical Society (EACS) Guidelines version 12.1 [7] and the U.S. Department of Health and Human Services (DHHS) Panel on Antiretroviral Guidelines for Adults and Adolescents [8]. For the guideline-based group, two infectious disease specialists independently selected representative questions covering key topics such as diagnosis, treatment, and prevention. In the third group, the questions were prepared from frequently asked questions on social media platforms (Google, X [formerly named Twitter], Facebook, YouTube). The terms “human immunodeficiency virus”, “HIV”, “acquired immunodeficiency syndrome”, and “AIDS” were searched online. The most frequently asked and recurrent questions were selected to represent common public concerns. In this study, questions about personal information, repetitive questions, questions with unclear answers, unrealistic questions, and questions with grammatical errors were excluded. Of the questions, 44.2% (*n* = 69) were prepared from the CDC, 30.8% (*n* = 48) were prepared from guidelines, and 25.0% (*n* = 39) were prepared from social media. The questions were categorized into four subgroups: “General information”, “Transmission”, “Diagnosis” and “Prevention and Treatment”.

Two specialists in infectious diseases and clinical microbiology (M.S.T. and M.S.O.) assessed the answers separately. A third infectious diseases and clinical microbiology specialist (Y.E.O.) reviewed responses that did not agree between the two specialists. All three experts are certified in the specialty of HIV. The scores for the responses that disagreed with the three specialists were evaluated jointly. The final score was determined by complete agreement. The reviewers used a scoring system from 1 to 4 points to assess the quality and reliability of the answers given by ChatGPT and Gemini. The scoring according to the adequacy and quality of the answers was as follows:

1. point: An answer that was completely incorrect or irrelevant.
2. points: An answer that was partially correct but includes some misleading information.
3. points: An answer that was generally correct but did not have sufficient detail.
4. points: An answer that was completely correct and sufficient.

The scores given by the reviewers based on ChatGPT and Gemini’s answers are listed in Supplementary Tables 1 and 2, respectively.

The consistency of the answers given by AI to the same question at different times and on different computers was also evaluated. Repeatability was evaluated as positive if the answers given when the same question was asked on different computers received the same score. If the answer to the same question did not have the same score, repeatability was evaluated as negative. In this case, only the first response from the LLMs was scored. Ethics committee approval was not required because this study did not include patient data.

### Statistical analysis

Statistical Package for Social Sciences (SPSS) version 25.0 (IBM Corp., Armonk, NY, USA) was used for statistical analysis. Categorical variables were presented as numbers (n) and percentages (%). Continuous variables were not normally distributed and are therefore presented as median (interquartile range, IQR). Differences between groups for continuous variables were assessed using the Mann–Whitney U test. Categorical variables were compared using the Chi-square test. Spearman’s correlation analysis was applied to investigate the relationship between the responses generated by ChatGPT and Gemini. To assess inter-rater reliability, Cohen’s kappa coefficient was calculated. A result with a p value < 0.05 was considered statistically significant.

## Results

The 156 questions included in the study were classified into four categories according to their topics: “General Information” (*n* = 43, 27.6%), “Transmission” (*n* = 31, 19.9%), “Diagnosis” (*n* = 18, 11.5%), and “Prevention and Treatment” (*n* = 64, 41.0%).

**Fig. 1:**
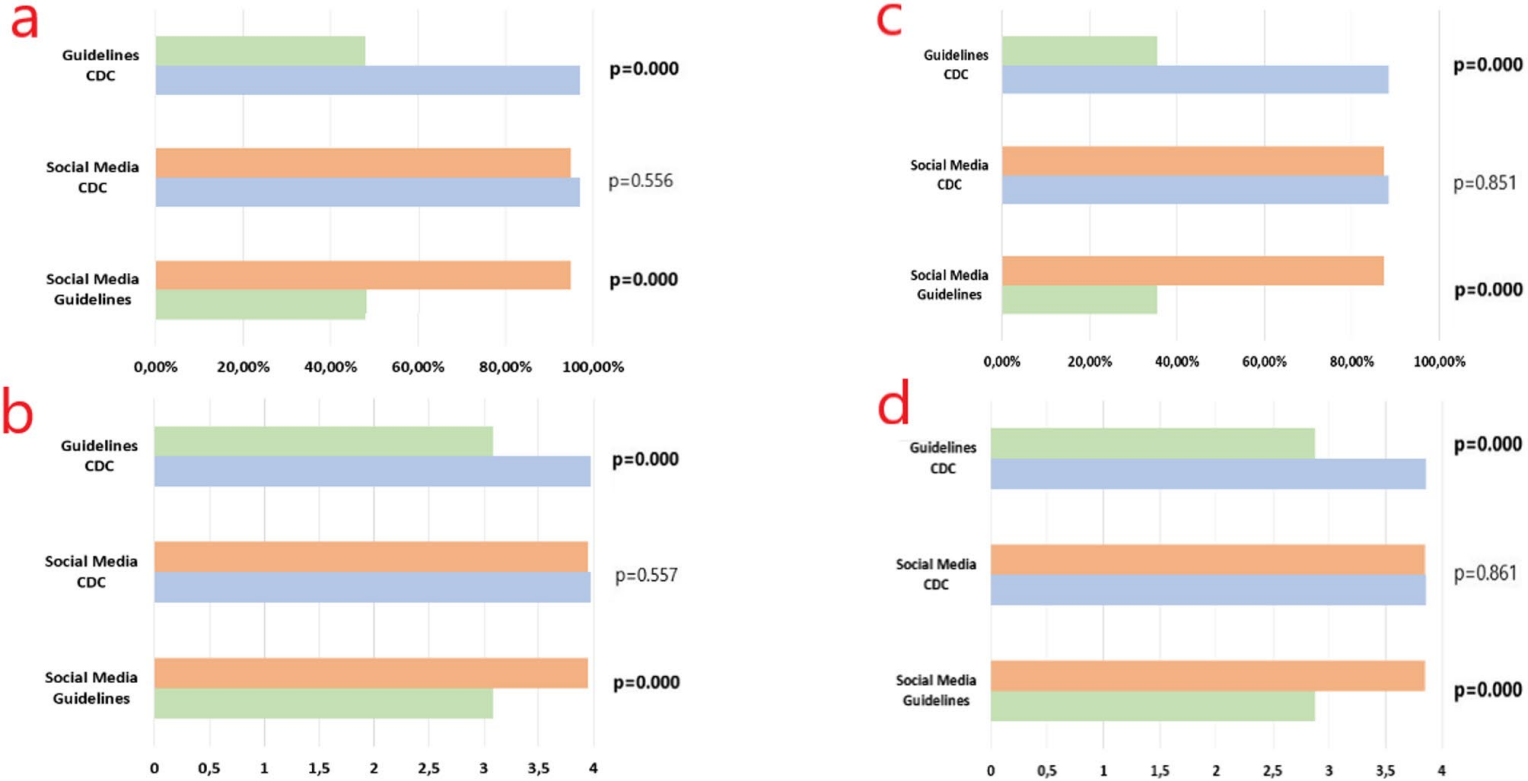
Comparison of the performance of ChatGPT and Gemini across question categories. Blue bars represent CDC questions, green bars represent guideline questions, and orange bars represent social media questions. (**a**) Comparison of the completely correct answer rates of ChatGPT among the main groups. (**b**) Comparison of the mean scores of ChatGPT answers among the main groups. (**c**) Comparison of the completely correct answer rates of Gemini among the main groups. (**d**) Comparison of the mean scores of Gemini answers among the main groups

The median score (IQR) and the mean score of the answers generated by ChatGPT for all questions were 4.00 (0.00) and 3.69 ± 0.72, respectively. The median score (IQR) of the answers to the guideline questions was significantly lower in comparison to both the CDC questions [3.00 (2.00) vs. 4.00 (0.00), *p* < 0.001] and the social media questions [3.00 (2.00) vs. 4.00 (0.00), *p* < 0.001]. Figure 1 shows a comparison of the mean scores for the Guideline questions, CDC questions, and social media questions. While the median (IQR) value for the topics of “General Information,” “Transmission,” and “Diagnosis” was 4.00 (0.00), the median (IQR) score for the topic of “Prevention and Treatment” was 4.00 (1.00). When the scores for the answers were compared by topics, the “Prevention and Treatment” topic was found to be lower than the others (vs. “General information,” *p* = 0.006; vs. “Transmission,” *p* = 0.001; and vs. “Diagnosis,” *p* = 0.049).

ChatGPT answered 81.4% of all questions completely correctly, 9.6% correctly but inadequately, 5.8% misleadingly, and 3.2% completely incorrectly. The distribution of the scores given to the answers according to the main groups and the topics is shown in Table 1. The rate of completely correct answers was lower for the guideline questions than for the CDC questions (47.9% vs. 97.1%, *p* < 0.001, OR = 37.0, 95% CI:8.00-166.7) and the social media questions (47.9% vs. 94.9%, *p* < 0.001, OR = 20.1, 95% CI: 4.35-93.0) (Fig. 1). The rate of completely correct answers to the questions about “Prevention and Treatment” was significantly lower than to the questions about “General Information” (67.2% vs. 88.4%, *p* = 0.012, OR = 3.72, 95% CI: 1.28–10.8) and “Transmission” (67.2% vs. 96.8%, *p* = 0.001, OR = 14.7, 95% CI: 1.87–111.1). No significant difference was found between the other topics in terms of completely correct answer rates (*p* > 0.05).

**Table 1:** Distribution of the scores of the answers of both LLMs according to the main groups and the topics

		Median (IQR)	1-point *n* (%)	2-points *n* (%)	3-points *n* (%)	4-points *n* (%)	Total *n* (%)
	Topics	** CDC Questions**					
ChatGPT	General Information	4.00 (0.00)	0 (0)	0 (0)	0 (0)	20 (100)	20 (100)
	Transmission	4.00 (0.00)	0 (0)	0 (0)	0 (0)	25 (100)	25 (100)
	Diagnosis	4.00 (0.00)	0 (0)	0 (0)	2 (15.4)	11 (84.6)	13 (100)
	Prevention and Treatment	4.00 (0.00)	0 (0)	0 (0)	0 (0)	11 (100)	11 (100)
	Total	4.00 (0.00)	0 (0)	0 (0)	2 (2.9)	67 (97.1)	69 (100)
		**Guideline Questions**					
	General Information	4.00 (1.00)	0 (0)	0 (0)	4 (40)	6 60)	10 (100)
	Prevention and Treatment	3.00 (2.00)	5 (13.2)	9 (23.7)	7 (18.4)	17 (44.7)	38 (100)
	Total	3.00 (2.00)	5 (10.4)	9 (18.8)	11 (22.9)	23 (47.9)	48 (100)
		**Social Media Questions**					
	General Information	4.00 (0.00)	0 (0)	0 (0)	1 (7.7)	12 (92.3)	13 (100)
	Transmission	4.00 (0.00)	0 (0)	0 (0)	1 (16.7)	5 (83.3)	6 (100)
	Diagnosis	4.00 (0.00)	0 (0)	0 (0)	0 (0)	5 (100)	5 (100)
	Prevention and Treatment	4.00 (0.00)	0 (0)	0 (0)	0 (0)	15 (100)	15 (100)
	Total	4.00 (0.00)	0 (0)	0 (0)	2 (5.1)	37 (94.9)	39 (100)
	All Questions	4.00 (0.00)	**5 (3.2)**	**9 (5.8)**	**15 (9.6)**	**127 (81.4)**	**156 (100)**
Gemini		**CDC Questions**					
	General Information	4.00 (0.00)	0 (0)	0 (0)	1 (5.0)	19 (95.0)	20 (100)
	Transmission	4.00 (0.00)	0 (0)	1 (4.0)	2 (8.0)	22 (88.0)	25 (100)
	Diagnosis	4.00 (1.00)	0 (0)	1 (7.7)	2 (15.4)	10 (76.9)	13 (100)
	Prevention and Treatment	4.00 (0.00)	0 (0)	0 (0)	1 (9.1)	10 (90.9)	11 (100)
	Total	4.00 (0.00)	0 (0)	2 (2.9)	6 (8.7)	61 (88.4)	69 (100)
		**Guideline Questions**					
	General Information	4.00 (1.00)	0 (0)	0 (0)	4 (40)	6 60)	10 (100)
	Prevention and Treatment	3.00 (2.00)	5 (13.2)	13 (34.2)	9 (23.7)	11 (28.9)	38 (100)
	Total	3.00 (2.00)	5 (10.4)	13 (27.1)	13 (27.1)	17 (35.4)	48 (100)
		**Social Media Questions**					
	General Information	4.00 (0.00)	0 (0)	0 (0)	2 (15.4)	11 (84.6)	13 (100)
	Transmission	4.00 (0.00)	0 (0)	0 (0)	0 (0)	6 (100)	6 (100)
	Diagnosis	4.00 (2.00)	0 (0)	1 (20.0)	1 (20.0)	3 (60.0)	5 (100)
	Prevention and Treatment	4.00 (0.00)	0 (0)	0 (0)	1 (6.7)	14 (93.3)	15 (100)
	Total	4.00 (0.00)	0 (0)	1 (2.6)	4 (10.3)	34 (87.2)	39 (100)
	All Questions	4.00 (1.00)	**5 (3.2)**	**16 (10.3)**	**23 (14.7)**	**112 (71.8)**	**156 (100)**

CDC: Centers for Disease Control and Prevention

The median score (IQR) of the answers generated by Gemini for all questions was 4.00 (1.00). The median score (IQR) for the guideline questions was found to be significantly lower than that for the CDC questions [3.00 (2.00) vs. 4.00 (0.00), *p* < 0.001] and the social media questions [3.00 (2.00) vs. 4.00 (0.00), *p* < 0.001]. A pairwise comparison of the mean scores for the guideline questions, CDC questions, and social media questions is presented in Fig. 1. The median score (IQR) of the answers to the “Prevention and Treatment” questions [4.00 (2.00)] was significantly lower than those for the “General Information” [4.00 (0.00)] and “Transmission” [4.00 (0.00)] questions (*p* < 0.001). There was no significant difference in mean scores between the other topics (*p* > 0.05).

Gemini answered 71.8% of all questions completely correctly, 14.7% correctly but inadequately, 10.3% misleadingly, and 3.2% completely incorrectly (Table 1). The rate of completely correct answers was lower for the guideline questions than for CDC questions (35.4% vs. 88.4%, *p* < 0.001, OR = 13.9, 95% CI:5.41–35.7) and the social media questions (35.4% vs. 87.2%, *p* < 0.001, OR = 12.4, 95% CI: 4.09–37.6) (Fig. 1). The rate of completely correct answers to the questions on “Prevention and Treatment” was significantly lower than to the questions on “General information” (54.7% vs. 83.7%, *p* = 0.002, OR = 4.26, 95% CI: 1.65–11.0) and “Transmission” (54.7% vs. 90.3%, *p* = 0.001, OR = 7.75, 95% CI: 2.13–27.8). There was no significant difference between the other topics in terms of the rate of completely correct answers (*p* > 0.05).

The median score (IQR) of ChatGPT’s answers was higher than that of Gemini for all HIV-related questions, although this was not statistically significant [4.00 (0.00) vs. 4.00 (1.00), *p* = 0.051]. ChatGPT answered the CDC questions with a higher score than Gemini [4.00 (0.00) vs. 4.00 (0.00), *p* = 0.048]. According to other main groups and the topics, there was no statistically significant difference between ChatGPT and Gemini (Table 2).

**Table 2:** Comparison of the mean scores of the answers of both LLMs according to the main groups and the topics

		*n*	Median (IQR) / Mean Score ± SD		*p*
			ChatGPT	Gemini	
	All Questions	156	4.00 (0.00) / 3.69 ± 0.72	4.00 (1.00) / 3.55 ± 0.81	0.051
Main Groups	CDC Questions	69	4.00 (0.00) / 3.97 ± 0.17	4.00 (0.00) / 3.86 ± 0.43	**0.048**
	Guideline Questions	48	3.00 (2.00) / 3.08 ± 1.05	3.00 (2.00) / 2.88 ± 1.02	0.272
	Social Media Questions	39	4.00 (0.00) / 3.95 ± 0.22	4.00 (0.00) / 3.85 ± 0.43	0.230
Topics	General Information	43	4.00 (0.00) / 3.88 ± 0.32	4.00 (0.00) / 3.84 ± 0.37	0.536
	Transmission	31	4.00 (0.00) / 3.97 ± 0.18	4.00 (0.00) / 3.87 ± 0.43	0.298
	Diagnosis	18	4.00 (0.00) / 3.89 ± 0.32	4.00 (1.00) / 3.61 ± 0.70	0.183
	Prevention and Treatment	64	4.00 (1.00) / 3.38 ± 1.00	4.00 (2.00) / 3.19 ± 1.02	0.206

CDC: Centers for Disease Control and Prevention

ChatGPT had a higher rate than Gemini in giving completely correct answers to all questions (81.4% vs. 71.8%, *p* = 0.045, OR = 1.72, 95% CI: 1.01–2.93). Also, in CDC questions, ChatGPT had a higher rate of correct answers than Gemini (97.1% vs. 88.4%, *p* = 0.049, OR = 4.39, 95% CI: 0.90–21.3). There was no significant difference between the two AIs in terms of completely correct answers according to the other main groups and the topics (Table 3).

**Table 3:** Comparison of completely correct answer rates of both LLMs according to the main groups and the topics

		*n*	Completely Correct Answer Rates (%)		OR	%95 CI	*p*
			ChatGPT	Gemini			
	All Questions	156	81.4	71.8	1.72	1.01–2.93	**0.045**
Main Groups	CDC Questions	69	97.1	88.4	4.39	0.90–21.3	**0.049**
	Guideline Questions	48	47.9	35.4	1.68	0.74–3.80	0.214
	Social Media Questions	39	94.9	87.2	2.72	0.49–14.93	0.235
Topics	General Information	43	88.4	83.7	1.48	0.43–5.08	0.534
	Transmission	31	96.8	90.3	3.22	0.32–32.3	0.301
	Diagnosis	18	88.9	72.2	6.15	0.051–18.5	0.206
	Prevention and Treatment	64	67.2	54.7	1.70	0.83–3.47	0.147

CDC: Centers for Disease Control and Prevention

There was a high positive correlation between ChatGPT and Gemini answers (*r* = 0.680, *p* < 0.001). According to Cohen’s kappa test, there was a substantial inter-rater agreement for ChatGPT (κ = 0.674) and Gemini (κ = 0.768). The reproducibility rate for ChatGPT was 94.8%, while that for Gemini was 90.3% (*p* = 0.135).

## Discussion

Although LLMs provide easy access to information on many topics, there is a significant disadvantage that these LLMs also contain false information. Misinformation about HIV can influence the decision-making processes and behaviours of non-experts, potentially having a negative impact on public health. While several studies have examined LLMs in the context of HIV, this area remains a limited field of research. For instance, one study explored a limited set of commonly asked patient questions regarding HIV and PrEP, assessing responses exclusively from ChatGPT [9]. Another study evaluated four different LLMs, but it also included questions on non-HIV infectious diseases and focused on analyzing language discrepancies rather than accuracy in answering the questions [10]. A third study assessed ChatGPT’s performance on HIV-related questions, but it did not specifically address guideline- or treatment-focused questions [11]. In addition, a recent study compared three LLMs in terms of accuracy, readability, and reliability for HIV education [12]; however, it only considered general HIV questions, leaving a gap regarding performance on more complex or guideline-based content. Zhao et al. also compared four LLMs (ChatGPT-4o, Gemini, Copilot, and Claude) on HIV-related questions; however, the number of questions evaluated was small, limiting generalizability [13]. Our study primarily aimed to compare the performance of ChatGPT and Gemini on HIV-related questions. Additionally, we examined differences in accuracy between general public questions and those focusing on guidelines and treatment, revealing a substantial drop in accuracy for more complex queries.

In our study, ChatGPT provided a higher rate of completely correct answers to HIV-related questions than Gemini (81.3% vs. 71.8%). Both AI applications responded to the CDC and the social media questions with high accuracy rates. However, the lowest accuracy rates were found in the guideline questions (ChatGPT 47.9%, Gemini 35.4%) and questions on “Prevention and Treatment” (ChatGPT 67.2%, Gemini 57.4%). Consistent with our findings, De Vito et al. evaluated ChatGPT for HIV prevention communication and reported that it answered 88.4% of the questions “almost completely correct” or “entirely correct” [11]. Similarly, Zhao et al. compared four LLMs on a limited set of HIV questions, reporting generally high accuracy [13]. Additionally, our findings revealed no significant difference in overall response scores between ChatGPT and Gemini, which aligns with Korkmaz et al.‘s findings, who also reported no significant difference between the two AI models. In that study, responses were rated on a five-point accuracy scale, with Gemini Advanced 2.0 Flash (4.31 ± 0.50) and ChatGPT-4o (4.29 ± 0.49) both demonstrating high accuracy [12].

The quality of AI-generated healthcare outputs varies: although promising results have been reported, the accuracy is inconsistent and requires careful validation. A growing body of research has demonstrated that LLMs can achieve high accuracy in various medical contexts. For example, a meta-analysis of 45 studies on ChatGPT in medical licensing exams reported an overall accuracy of 81%, with ChatGPT-4 exceeding average medical student scores in multiple cases [14]. Similarly, ChatGPT-4 showed 95.9% agreement with physicians in differential diagnosis assessments [15], and strong performance in radiology and other clinical areas [16–18]. However, limitations remain. In assessments of clinical reasoning and emergency care scenarios, AI models—including ChatGPT-4 and Gemini—sometimes underperformed relative to experienced clinicians, with occasional provision of misleading or potentially harmful information [19–21]. In our study, ChatGPT and Gemini provided completely wrong or misleading answers to 9.0% and 13.5% of the questions, respectively. Answers containing incorrect information is one of the important limitations of AI applications and improvements are needed in this regard.

While LLMs generally answer frequently asked questions and social media–derived questions with high accuracy, their performance declines on more complex issues such as guideline- and treatment-related questions. For example, ChatGPT correctly answered 92% of social media questions but only 69% of guideline questions across infectious disease topics [22], and showed similar patterns for cervical cancer [23] and osteoporosis [24]. Performance also decreased for complex dermatological cases [25]. In our study, both ChatGPT and Gemini exhibited notably lower accuracy on guideline and “Prevention and Treatment” questions. Similar to other medical topics, our findings show a decline in accuracy for complex questions, expand upon previous HIV-related research, and suggest that users should not rely solely on LLM-generated information for guidance or treatment-specific decisions.

The performances of different AI applications in healthcare have been compared in several studies. In many studies, different versions of ChatGPT (3.5, 4o, and 4.0) demonstrated better performance than Gemini [26–28]. In some studies, ChatGPT-4 performed better than Gemini and ChatGPT-3.5, while ChatGPT-3.5 and Gemini showed similar performance [29, 30]. Similarly, in another study comparing ChatGPT-3.5 and Gemini, both LLMs showed similar performance [31]. In our study, the completely correct response rate was significantly higher in the responses produced by ChatGPT-4o than Gemini. However, there was no significant difference between the mean scores of the responses produced by both LLMs.

The reproducibility of the information provided by LLMs is as important as reliability and accuracy, because it is very difficult to generalize study findings on platforms where repeatability is low. In existing studies in the literature, reproducibility rates for ChatGPT have been reported to be between 70% and 100% [23, 24, 32], and for Gemini between 50% and 92% [33, 34]. In our study, the repeatability rates of the answers were found to be similarly high for ChatGPT and Gemini, 94.8% and 90.3%, respectively.

While the main objective of our study was to compare the performance of ChatGPT and Gemini on HIV-related questions, we also conducted an additional analysis examining differences in accuracy between general public questions and questions on clinical management and guideline-based content, providing new insights into LLM performance in more complex, guideline-focused scenarios. However, our study had several limitations. Firstly, it should be noted that the questions used in this study represent only a fraction of the total number of questions that could be posed within this field of inquiry. Secondly, while expert assessment inherently involves a degree of subjectivity, any disagreement between the two primary evaluators was resolved by a third expert, thus minimizing subjectivity. Thirdly, this study did not assess the clarity and understandability of the LLMs’ answers for non-expert users, which may affect how easily individuals can interpret and apply the information provided.

## Conclusion

In this study, ChatGPT and Gemini answered CDC- and social media–based HIV questions with high accuracy, but both models showed substantially lower accuracy for guideline-based questions and for topics related to prevention and treatment. These findings suggest that while LLMs may be useful for rapid access to general information and for patient-directed educational purposes. However, caution should be exercised regarding the reliability of AI-generated outputs for complex clinical decisions, such as treatment and disease management, since these models generate responses probabilistically rather than retrieving verified guidelines.

## Electronic Supplementary Material

Below is the link to the electronic supplementary material.

Supplementary Material 1

Supplementary Material 2

## Data Availability

The datasets analysed during the current study are available from the corresponding author on reasonable request.

## Abbreviations

AI: Artificial intelligence
CDC: Centers for Disease Control and Prevention
ChatGPT: Chat Generative Pre-trained Transformer
DHHS: United States Department of Health and Human Services
EACS: European AIDS Clinical Society
HIV: Human Immunodeficiency Virus

## References

[1] Global HIV. Oct & AIDS statistics — Fact sheet | UNAIDS [Internet]. https://www.unaids.org/en/resources/fact-sheet. Accessed 11 2025.

[2] Trickey A, Sabin CA, Burkholder G, Crane H, d’Arminio Monforte A, Egger M, et al. Life expectancy after 2015 of adults with HIV on long-term antiretroviral therapy in Europe and North america: a collaborative analysis of cohort studies. Lancet HIV. 2023. 10.1016/S2352-3018(23)00028-0. 36958365 10.1016/S2352-3018(23)00028-0 PMC10288029

[3] Obermeyer Z, Emanuel EJ. Predicting the Future — Big Data, machine Learning, and clinical medicine. N Engl J Med. 2016. 10.1056/NEJMp1606181. 27682033 10.1056/NEJMp1606181 PMC5070532

[4] Rashid A, Bin, Kausik MAK. AI revolutionizing industries worldwide: A comprehensive overview of its diverse applications. Hybrid Adv. 2024. 10.1016/J.HYBADV.2024.100277.

[5] Introducing GPT- 4. o and more tools to ChatGPT free users | OpenAI [Internet]. 2025. https://openai.com/index/gpt-4o-and-more-tools-to-chatgpt-free/. Accessed 10 Jun 2025.

[6] Gemini [Internet]. 2025. https://gemini.google.com/app. Accessed 10 Jun 2025.

[7] EACS Guidelines. 2024 — EACS Guidelines [Internet]. https://eacs.sanfordguide.com/. Accessed 12 Nov 2024.

[8] HIV/AIDS Treatment Guidelines. | Clinicalinfo.HIV.gov [Internet]. https://clinicalinfo.hiv.gov/en/guidelines. Accessed 12 Nov 2024.

[9] Ganicho J, Carlos M, Cristóvão G, Cruz C, Leal M, Garrote AR, et al. Use of ChatGPT in HIV infection counselling and literacy. Acta Med Port. 2025. 10.20344/amp.22805. 40580431 10.20344/amp.22805

[10] Sallam M, Al-Mahzoum K, Alshuaib O, Alhajri H, Alotaibi F, Alkhurainej D, et al. Language discrepancies in the performance of generative artificial intelligence models: an examination of infectious disease queries in english and Arabic. BMC Infect Dis. 2024. 10.1186/s12879-024-09725-y. 39118057 10.1186/s12879-024-09725-y PMC11308449

[11] De Vito A, Colpani A, Moi G, Babudieri S, Calcagno A, Calvino V, et al. Assessing chatgpt’s potential in HIV prevention communication: A comprehensive evaluation of Accuracy, Completeness, and inclusivity. AIDS Behav. 2024. 10.1007/s10461-024-04391-2. 38836986 10.1007/s10461-024-04391-2 PMC11286632

[12] Eren Korkmaz Ö, Açıkalın Arıkan B, Sayın Kutlu S, Kaptan Aydoğmuş F, Sezak N. Artificial intelligence Meets HIV education: comparing three large Language models on accuracy, readability, and reliability. Int J STD AIDS. 2025. 10.1177/09564624251372369. 40905356 10.1177/09564624251372369

[13] Zhao CY, Song C, Yang T, Huang AC, Qiang HB, Gong CM, et al. AI-Driven large Language models in health consultations for HIV patients. J Multidiscip Healthc. 2025. 10.2147/JMDH.S533621. 40895329 10.2147/JMDH.S533621 PMC12396217

[14] Liu M, Okuhara T, Chang XY, Shirabe R, Nishiie Y, Okada H, et al. Performance of ChatGPT across different versions in medical licensing examinations worldwide: systematic review and Meta-Analysis. J Med Internet Res. 2024. 10.2196/60807. 39052324 10.2196/60807 PMC11310649

[15] Mizuta K, Hirosawa T, Harada Y, Shimizu T. Can ChatGPT-4 evaluate whether a differential diagnosis list contains the correct diagnosis as accurately as a physician? Diagnosis. 2024. 10.1515/dx-2024-0027. 38465399 10.1515/dx-2024-0027

[16] Keshavarz P, Bagherieh S, Nabipoorashrafi SA, Chalian H, Rahsepar AA, Kim GHJ, et al. ChatGPT in radiology: A systematic review of performance, pitfalls, and future perspectives. Diagn Interv Imaging. 2024. 10.1016/j.diii.2024.04.003. 38679540 10.1016/j.diii.2024.04.003

[17] Musheyev D, Pan A, Kabarriti AE, Loeb S, Borin JF. Quality of information about kidney stones from artificial intelligence chatbots. J Endourol. 2024. 10.1089/end.2023.0484. 39001821 10.1089/end.2023.0484

[18] King RC, Samaan JS, Yeo YH, Peng Y, Kunkel DC, Habib AA, et al. A multidisciplinary assessment of chatgpt’s knowledge of amyloidosis: observational study. JMIR Cardio. 2024. 10.2196/53421. 38640472 10.2196/53421 PMC11069089

[19] Maillard A, Micheli G, Lefevre L, Guyonnet C, Poyart C, Canouï E, et al. Can chatbot artificial intelligence replace infectious diseases physicians in the management of bloodstream infections? A prospective cohort study. Clin Infect Dis. 2024. 10.1093/cid/ciad632. 37823416 10.1093/cid/ciad632

[20] Yau JYS, Saadat S, Hsu E, Murphy LSL, Roh JS, Suchard J, et al. Accuracy of prospective assessments of 4 large Language model chatbot responses to patient questions about emergency care: experimental comparative study. J Med Internet Res. 2024. 10.2196/60291. 39496149 10.2196/60291 PMC11574488

[21] Jeblick K, Schachtner B, Dexl J, Mittermeier A, Stüber AT, Topalis J, et al. ChatGPT makes medicine easy to swallow: an exploratory case study on simplified radiology reports. Eur Radiol. 2023. 10.1007/s00330-023-10213-1. 37794249 10.1007/s00330-023-10213-1 PMC11126432

[22] Tunçer G, Güçlü KG. How reliable is ChatGPT as a novel consultant in infectious diseases and clinical microbiology? Inf Dis Clin Microbiol. 2024; 10.36519/idcm.2024.286. 10.36519/idcm.2024.286 PMC11020004 38633442

[23] Yurtcu E, Ozvural S, Keyif B. Analyzing the performance of ChatGPT in answering inquiries about cervical cancer. Int J Gynecol Obstet. 2024. 10.1002/ijgo.15861. 10.1002/ijgo.15861 PMC11726164 39148482

[24] Cinar C. Analyzing the performance of ChatGPT about osteoporosis. Cureus. 2023. 10.7759/cureus.45890. 37885522 10.7759/cureus.45890 PMC10599213

[25] Goktas P, Grzybowski A. Assessing the impact of ChatGPT in dermatology: A comprehensive rapid review. J Clin Med. 2024. 10.3390/jcm13195909. 39407969 10.3390/jcm13195909 PMC11477344

[26] Carlà MM, Gambini G, Baldascino A, Giannuzzi F, Boselli F, Crincoli E, et al. Exploring AI-chatbots’ capability to suggest surgical planning in ophthalmology: ChatGPT versus Google gemini analysis of retinal detachment cases. Br J Ophthalmol. 2024. 10.1136/bjo-2023-325143. 38448201 10.1136/bjo-2023-325143

[27] Rossettini G, Rodeghiero L, Corradi F, Cook C, Pillastrini P, Turolla A, et al. Comparative accuracy of ChatGPT-4, Microsoft copilot and Google gemini in the Italian entrance test for healthcare sciences degrees: a cross-sectional study. BMC Med Educ. 2024. 10.1186/s12909-024-05630-9. 38926809 10.1186/s12909-024-05630-9 PMC11210096

[28] Abdul Sami M, Abdul Samad M, Parekh K, Suthar PP. Comparative accuracy of ChatGPT 4.0 and Google gemini in answering pediatric radiology Text-Based questions. Cureus. 2024. 10.7759/cureus.70897. 39497868 10.7759/cureus.70897 PMC11534303

[29] Toyama Y, Harigai A, Abe M, Nagano M, Kawabata M, Seki Y, et al. Performance evaluation of ChatGPT, GPT-4, and bard on the official board examination of the Japan radiology society. Jpn J Radiol. 2024. 10.1007/s11604-023-01491-2. 37792149 10.1007/s11604-023-01491-2 PMC10811006

[30] Khan AA, Yunus R, Sohail M, Rehman TA, Saeed S, Bu Y, et al. Artificial intelligence for anesthesiology Board-Style examination questions: role of large Language models. J Cardiothorac Vasc Anesth. 2024. 10.1053/j.jvca.2024.01.032. 38423884 10.1053/j.jvca.2024.01.032

[31] Doğan L, Özçakmakcı GB, Yılmaz ĬE. The performance of chatbots and the AAPOS website as a tool for amblyopia education. J Pediatr Ophthalmol Strabismus. 2024. 10.3928/01913913-20240409-01. 38661309 10.3928/01913913-20240409-01

[32] Ozgor BY, Simavi MA. Accuracy and reproducibility of chatgpt’s free version answers about endometriosis. Int J Gynecol Obstet. 2024. 10.1002/ijgo.15309. 10.1002/ijgo.15309 38108232

[33] Iannantuono GM, Bracken-Clarke D, Karzai F, Choo-Wosoba H, Gulley JL, Floudas CS. Comparison of large Language models in answering Immuno-Oncology questions: A Cross-Sectional study. Oncologist. 2024. 10.1093/oncolo/oyae009. 38309720 10.1093/oncolo/oyae009 PMC11067804

[34] Sahin Ozdemir M, Ozdemir YE. Comparison of the performances between ChatGPT and gemini in answering questions on viral hepatitis. Sci Rep. 2025. 10.1038/s41598-024-83575-1. 39799203 10.1038/s41598-024-83575-1 PMC11724965

