# Supplementary material for: Comparison of the accuracy and reliability of ChatGPT-4o and Gemini in answering HIV-related questions
Source: BMC Infect Dis. 2025 Nov 17;25:1591. doi: 10.1186/s12879-025-12022-x (PMC12625381; doi:10.1186/s12879-025-12022-x)
Supplement: Supplementary file 2 — Supplementary Material 2 [file 12879_2025_12022_MOESM2_ESM.docx]

**Supplementary Table 2.** Scores for answers generated by Gemini according to the reviewers

| **Questions** | | **Specialist 1** | **Specialist 2** | **Specialist 3** |
| --- | --- | --- | --- | --- |
| **CDC Questions - General information** | | | | |
| **1** | Is there a cure for HIV? | 4 | 4 |  |
| **2** | Does HIV remain in the body for life? | 4 | 4 |  |
| **3** | Can HIV be controlled with medication? | 4 | 4 |  |
| **4** | How can I find out if I have HIV or not? | 4 | 4 |  |
| **5** | Which symptoms occur after HIV infection? | 4 | 4 |  |
| **6** | Can HIV survive outside the human body? | 4 | 4 |  |
| **7** | What is the HIV viral load? | 4 | 4 |  |
| **8** | What is the undetectable viral load? | 4 | 4 |  |
| **9** | Does a high viral load increase the risk of HIV infection? | 4 | 4 |  |
| **10** | What are the stages of HIV disease? | 4 | 4 |  |
| **11** | At which stages of HIV disease is the viral load high? | 4 | 4 |  |
| **12** | Do alcohol and drug use increase the risk of HIV infection? | 4 | 4 |  |
| **13** | What is AIDS? | 3 | 3 |  |
| **14** | Approximately how long do AIDS patients live without treatment? | 4 | 4 |  |
| **15** | What should I do if I test positive for HIV? | 4 | 4 |  |
| **16** | If my HIV test is negative, am I definitely not infected with HIV? | 4 | 4 |  |
| **17** | Who should I tell if my HIV test is positive? | 3 | 4 | 4 |
| **18** | What's the CD4 count? | 4 | 4 |  |
| **19** | Does HIV disease reduce the CD4 count? | 4 | 4 |  |
| **20** | What does ‘Undetectable = Untransmittable’ mean? | 4 | 4 |  |
| **CDC Questions – Transmission** | | | | |
| **21** | How is HIV spread? | 3 | 3 |  |
| **22** | Which body fluids HIV spreads from? | 4 | 4 |  |
| **23** | Which ways is HIV most commonly spread? | 4 | 4 |  |
| **24** | Can HIV be transmitted to the baby during pregnancy? | 4 | 4 |  |
| **25** | Can HIV be transmitted through breastfeeding? | 4 | 4 |  |
| **26** | Can HIV be transmitted through kissing? | 4 | 4 |  |
| **27** | Can HIV be transmitted through touch? | 4 | 4 |  |
| **28** | Which sexual activities can transmit HIV? | 4 | 4 |  |
| **29** | What sexual activity is most likely to transmit HIV? | 4 | 4 |  |
| **30** | Can HIV be transmitted through oral sex? | 4 | 4 |  |
| **31** | Can HIV be transmitted through vaginal sex? | 4 | 4 |  |
| **32** | Can HIV be transmitted through anal sex? | 4 | 4 |  |
| **33** | Can HIV be transmitted with used syringes? | 4 | 4 |  |
| **34** | Can HIV be transmitted by hugging and shaking hands? | 4 | 4 |  |
| **35** | Can HIV be transmitted through the air? | 4 | 4 |  |
| **36** | Can HIV be transmitted through shared toilet use? | 4 | 4 |  |
| **37** | Can HIV be transmitted through sweat, saliva and tears? | 4 | 4 |  |
| **38** | Can HIV be transmitted by insect, mosquito or tick bites? | 4 | 4 |  |
| **39** | Can HIV be transmitted through sharing food dishes? | 4 | 4 |  |
| **40** | Can HIV be transmitted through tattoos, body piercings and cosmetic procedures? | 2 | 3 | 3 |
| **41** | The most common ways in which HIV is transmitted to children? | 4 | 4 |  |
| **42** | Can I get HIV when donating blood? | 4 | 4 |  |
| **43** | Can someone who has an undetected viral load transmit HIV through sex? | 2 | 2 |  |
| **44** | Does the presence of another sexually transmitted disease facilitate HIV transmission? | 4 | 4 |  |
| **45** | Can HIV patients protect their partners from the disease with medication? | 3 | 4 | 4 |
| **CDC Questions – Diagnosis** | | | | |
| **46** | When should I be tested for HIV? | 4 | 4 |  |
| **47** | Who should be tested for HIV more frequently? | 3 | 3 |  |
| **48** | Do gay and bisexual men need more frequent HIV tests? | 4 | 4 |  |
| **49** | How often should gay and bisexual men be tested for HIV? | 4 | 4 |  |
| **50** | Should pregnant women have an HIV test? | 4 | 4 |  |
| **51** | How can I take an HIV test? | 3 | 4 | 3 |
| **52** | Can I take an HIV self-test? | 4 | 4 |  |
| **53** | How is the fastest HIV test done? | 3 | 4 | 4 |
| **54** | What is the window period for HIV diagnosis? | 2 | 2 |  |
| **55** | Which test is positive at the earliest after HIV infection? | 4 | 4 |  |
| **56** | On how many days after infection is the HIV test positive at the earliest? | 4 | 4 |  |
| **57** | On how many days after HIV infection do rapid antigen/antibody tests become positive at the earliest? | 4 | 4 |  |
| **58** | On how many days after HIV infection do nucleic acid tests become positive at the earliest? | 4 | 4 |  |
| **CDC Questions - Prevention and Treatment** | | | | |
| **59** | What are the ways to prevent HIV? | 4 | 4 |  |
| **60** | Does using a condom during sex protect against HIV? | 4 | 4 |  |
| **61** | In which conditions should pre-exposure prophylaxis be performed to protect against HIV? | 4 | 4 |  |
| **62** | Should I take an HIV test before taking pre-exposure prophylaxis? | 4 | 4 |  |
| **63** | Is there any way for an HIV-positive person to prevent sexual transmission? | 4 | 4 |  |
| **64** | When should HIV treatment be initiated? | 3 | 4 | 3 |
| **65** | What treatments are there for HIV? | 4 | 4 |  |
| **66** | What are the benefits of HIV treatment? | 4 | 4 |  |
| **67** | When does HIV drug resistance occur? | 4 | 4 |  |
| **68** | What if I don't take my HIV medication regularly? | 4 | 4 |  |
| **69** | What side effects does HIV treatment have? | 4 | 4 |  |
| **Guidelines Questions - General information** | | | | |
| **70** | Who is an HIV controller? | 4 | 4 |  |
| **71** | When can HIV-RNA be detected in the blood after infection? | 4 | 4 |  |
| **72** | Which other diseases should patients diagnosed with HIV also be screened for? | 3 | 4 | 3 |
| **73** | Can HIV patients breastfeed? | 4 | 4 |  |
| **74** | How often should viral load be tested in pregnant HIV patients? | 4 | 4 |  |
| **75** | Can an HIV patient get a live vaccine? | 3 | 2 | 3 |
| **76** | Which vaccinations should HIV patients have? | 3 | 2 | 3 |
| **77** | Can an HIV patient receiving treatment sexually transmit HIV to others? | 4 | 4 |  |
| **78** | Is depression more common in HIV patients? | 4 | 4 |  |
| **79** | Can HIV patients receive organ transplants? | 3 | 3 |  |
| **Guidelines Questions – Treatment** | | | | |
| **80** | Is the CD4 count important for the timing of initiation of antiretroviral therapy for HIV? | 2 | 2 |  |
| **81** | In which situations should antiretroviral therapy for HIV be started immediately? | 3 | 3 |  |
| **82** | What are the benefits of early treatment for HIV? | 4 | 4 |  |
| **83** | Should drug resistance testing be performed before HIV treatment? | 4 | 4 |  |
| **84** | What are the first-line regimens for HIV treatment? | 2 | 2 |  |
| **85** | Which treatments for HIV can cause weight increase? | 2 | 2 |  |
| **86** | In which cases should abacavir not be used to treatment HIV? | 3 | 4 | 3 |
| **87** | Can a single drug be used in HIV treatment? | 4 | 4 |  |
| **88** | What is virological failure in HIV treatment? | 3 | 4 | 3 |
| **89** | What is virological rebound in HIV treatment? | 3 | 3 |  |
| **90** | Can pregnant women take HIV treatment? | 4 | 4 |  |
| **91** | What are the first-line HIV treatment regimens for pregnant HIV patients? | 3 | 4 | 3 |
| **92** | When should antiretroviral therapy be started in HIV patients with a diagnosis of tuberculosis? | 3 | 2 | 2 |
| **93** | What are the first-line treatment regimens for HIV patients with a diagnosis of tuberculosis? | 2 | 2 |  |
| **94** | Which of the medicines used in HIV treatment can cause low vitamin D as a side effect? | 1 | 1 |  |
| **95** | How should pregnant women with HIV detected during labour be approached? | 3 | 3 |  |
| **96** | What should be the delivery strategy for pregnant women receiving HIV treatment? | 4 | 4 |  |
| **97** | What are the antiretroviral treatment options for HIV patients with hepatitis B? | 2 | 2 |  |
| **98** | When should antiretroviral therapy for HIV be started in the presence of opportunistic infection? | 1 | 1 |  |
| **99** | Which of the medicines used in HIV treatment can cause kidney side effects? | 3 | 3 |  |
| **100** | Which of the medicines used in HIV treatment can cause depression as a side effect? | 3 | 3 |  |
| **101** | Which of the medicines used in HIV treatment can impair lipid levels as a side effect? | 3 | 3 |  |
| **102** | Which of the medicines used in HIV treatment can cause ischaemic heart disease as a side effect? | 2 | 2 |  |
| **103** | Which of the tuberculosis drugs interacts more frequently with antiretroviral drugs? | 4 | 4 |  |
| **104** | Which of the antiretroviral drugs have an openable capsule form? | 1 | 1 |  |
| **105** | Which of the antiretroviral drugs does not require dose adjustment in liver dysfunction? | 2 | 2 |  |
| **106** | Which of the antiretroviral drugs do not require dose adjustment for patients receiving haemodialysis? | 2 | 2 |  |
| **107** | In what cases should people living with HIV be screened for bone disease? | 2 | 2 |  |
| **Guidelines Questions – Prevention** | | | | |
| **108** | When should prophylaxis be started after HIV exposure? | 4 | 4 |  |
| **109** | What is the duration of prophylaxis after HIV exposure? | 4 | 4 |  |
| **110** | In which cases is pre-exposure prophylaxis for HIV recommended? | 4 | 4 |  |
| **111** | How often should I be tested for HIV while receiving pre-exposure prophylaxis for HIV? | 4 | 4 |  |
| **112** | Should hepatitis B serology be tested before starting pre-exposure prophylaxis for HIV? | 2 | 2 |  |
| **113** | Does pre-exposure prophylaxis for HIV also protect against other sexually transmitted infections? | 4 | 4 |  |
| **114** | How is pre-exposure prophylaxis for HIV applied? | 2 | 2 |  |
| **115** | How is ‘on-demand’ pre-exposure prophylaxis for HIV implemented? | 1 | 1 |  |
| **116** | Can women receive ‘on demand’ pre-exposure prophylaxis for HIV? | 2 | 2 |  |
| **117** | I am taking pre-exposure prophylaxis for HIV. How many days after exposure should I stop taking it? | 1 | 2 | 1 |
| **Social Media Questions - General information** | | | | |
| **118** | Is HIV always deadly? | 4 | 4 |  |
| **119** | What is the difference between HIV-1 and HIV-2? | 4 | 4 |  |
| **120** | How long does HIV live outside the human body? | 4 | 4 |  |
| **121** | I have HIV, which department should I go to? | 4 | 4 |  |
| **122** | Does HIV cause cancer? | 4 | 4 |  |
| **123** | Can I donate blood if I have HIV? | 4 | 4 |  |
| **124** | Can I donate organs if I have HIV? | 4 | 4 |  |
| **125** | Can HIV positive individuals/couples have children? | 3 | 3 |  |
| **126** | What should I do and how should I behave if my friend tells me that he/she is HIV positive? | 4 | 4 |  |
| **127** | What is the life expectancy for someone receiving HIV treatment? | 4 | 4 |  |
| **128** | What is viral suppression? | 4 | 4 |  |
| **129** | What is a viral blip? | 4 | 4 |  |
| **130** | The coronavirus vaccine was available in as little as a year. But why is the HIV vaccine still not found? | 3 | 3 |  |
| **Social Media Questions - Transmission** | | | | |
| **131** | In which ways is HIV not transmitted? | 4 | 4 |  |
| **132** | Is HIV transmitted easily? | 4 | 4 |  |
| **133** | Can I get HIV from a used needle I picked up? | 4 | 4 |  |
| **134** | Can I get HIV from a swimming pool? | 4 | 4 |  |
| **135** | Can I get HIV from a mosquito bite? | 4 | 4 |  |
| **136** | Can HIV be transmitted from leeches? | 4 | 4 |  |
| **Social Media Questions - Diagnosis** | | | | |
| **137** | How is HIV diagnosed? | 3 | 4 | 3 |
| **138** | How long after suspected contact should I have an HIV test? | 2 | 2 |  |
| **139** | How to quickly find out if a person has HIV? | 4 | 4 |  |
| **140** | Are there factors that can influence the HIV test and lead to a false result? | 4 | 4 |  |
| **141** | Are self/home HIV tests reliable? | 4 | 4 |  |
| **Social Media Questions - Prevention and Treatment** | | | | |
| **142** | Is there a drug treatment for HIV and does it work? | 4 | 4 |  |
| **143** | Is there any method that can definitively prevent HIV? | 4 | 4 |  |
| **144** | I have an active sex life. Can I be protected from HIV? | 4 | 4 |  |
| **145** | I use a needle to inject medicine. Can I still be protected from HIV? | 4 | 4 |  |
| **146** | I found out that someone I love very much is HIV positive. Is there any precautions I should take to protect myself? | 4 | 4 |  |
| **147** | What is pre-exposure prophylaxis for HIV? | 4 | 2 | 4 |
| **148** | What is post-exposure prophylaxis for HIV? | 4 | 4 |  |
| **149** | Are condoms really effective in preventing HIV? | 4 | 4 |  |
| **150** | Can I use my friend's HIV treatment medicines as pre-exposure prophylaxis? | 4 | 4 |  |
| **151** | Does circumcision protect from HIV? | 4 | 4 |  |
| **152** | Is there a vaccine against HIV? | 4 | 4 |  |
| **153** | What happens if I stop HIV medication? | 4 | 4 |  |
| **154** | Is it a problem if I take my HIV medication for a certain period of time, for example 30 minutes or 1 hour late? | 3 | 3 |  |
| **155** | Why is there no definitive cure for HIV? | 4 | 4 |  |
| **156** | Can marrow transplantation be a definitive method for the definitive treatment of HIV? | 4 | 4 |  |

CDC: Centers for Disease Control and Prevention
